# An Essay on Curvatures and Distortions of the Spine, and Some Other Morbid Derangements to Which It Is Subject

**Published:** 1823-08

**Authors:** R. W. Bampfield

**Affiliations:** one of the Surgeons to the Royal Metropolitan Infirmary, &c.


THE LONDON
### Medical and Physical Journal.

2 OF VOL. L.] AUGUST, 1823. [No. 294.

For many fortunate discoveries in medicine, and for the detection of numerous errors, the world is indebted to the rapid circulation of Monthly Journals; and there never existed any work, to which the Faculty, in Europe and America, were under deeper obligations, than to the Medical and Physical Journal of London, now forming a long, but an invaluable, series.—RUSH.

### ORIGINAL COMMUNICATIONS,
### SELECT OBSERVATIONS, &c.

ART. I.—*An Essay on Curvatures and Distortions of the Spine, and some other Morbid Derangements to which it is subject.* By R. W. BAMPFIELD, Esq. one of the Surgeons to the Royal Metropolitan Infirmary, &c.

"What can we reason, but from what we know?"

POPE'S *Essay on Man.*

[Continued from page 187.]

IN the former parts of this essay, I have endeavoured to investigate some causes of distortions of the spine; and, in tracing out the connexion between causes and their consequences, to explain their *modus operandi.* I have also attempted to demonstrate, by example and analogical illustration, that a permanent curvature of the spine can only be effected by those structural changes in the horizontal surfaces of the bodies of the vertebræ, or of their intervertebral cartilages, which occasion such disproportions as render one side thinner than its opposite, or by such progressive destruction as leads to the partial or total removal of the portions of the vertebræ, or their inner line of curve. In the course of this demonstration and reasoning, the pathological fact of the absorption of the bodies and intervertebral cartilages of the spine from the effects of unnatural pressure, without the intervention or presence of caries, has been introduced to the public notice.

It is now proposed to take a view of those causes that have been, for the purpose of discussing them, admitted to be capable of producing temporary distortion or curvature. These are relaxation of the ligaments of the vertebræ, and diminished power of the muscles of the back.

In a series of communications to this Journal, Dr. E. Harrison has advanced an opinion, that not only "the common variety"* of curved spine, but all other instances, have their origin in the

* The *London Med. and Phys. Journal* for February 1821, page 110.

relaxation of the vertebral ligaments; an opinion which has excited considerable interest, and made some proselytes, from the alluring ease with which such a form of disease is said to be cured, and "*the spinal column made wholly to recover its natural form and powers.*"* Dr. H.'s notion is given at p. 106-7 of the Medical and Physical Journal for February 1821. In discussing "the true cause of spinal complaints," he observes, "I am of opinion that we shall find it in the connecting ligaments, 'which seem to have lost part of their power of holding the bones together.' These get relaxed, and suffer a single vertebra to become slightly displaced. The column now losing its natural firmness, other bones begin to press unduly upon the surrounding ligaments; they, in turn, get relaxed and elongated, by which *the dislocation* is increased, and the distortion permanently established. The direction becomes lateral, anterior, or posterior, according to circumstances; but *the malady has in every instance the same origin, and requires the same mode of cure.*" The manner in which the relaxation of ligaments is produced is thus stated: "The muscles destined to move the vertebræ are attached to the articulating fibrous structure. This is stretched by them, and in weakly habits becomes preternaturally elongated, partly by the muscular force pulling it, partly by the bones being pushed against it in the various turns and gesticulations of the body. As these parts gradually give way, the joints slowly enlarge, and admit of greater motion. A slight luxation is first produced in one joint, and afterwards in several."† The consequences of this relaxation of ligaments are said to be either "a luxation or subluxation of the vertebræ;" terms which necessarily correspond with those distinctions of luxations into complete and incomplete, so long of established use by the profession; and, from relaxation of ligaments allowing of different states and degrees of dislocation, Dr. H. attempts to account for all the varieties of curvature and distortions of the spine, and the symptoms accompanying them.‡ "According to this view of the subject, the obvious indication for the cure of spinal affections consists in restoring the displaced bones to their natural situations, that the spinal cord and its nerves, relieved from injurious pressure and disturbance, may be reinstated in their former abilities. In all the cases hitherto treated agreeably to these principles, the success has been complete."§

We profess to have been captivated by the facilities and cheering prospects which these doctrines offered of curing

* *London Med. and Phys. Journal* for February 1821, p. 109.
† *Ibid*, for November 1820, p. 371.
‡ *Ibid*, p. 368.
§ *Ibid*, for February 1821, p. 113.

some of the patients who presented at the Institution for Sick Children; and we still feel under obligations to Dr. H. for encouraging expectations of recovery by a deviation from the ordinary practice, although he has thought proper to conceal his own. Yet we proceeded with that degree of doubt which is so often the parent of truth, as there were certain expressions in his essay, whose precise meaning we could not distinctly comprehend: as when it is stated, that "the cervical bones disappeared" without informing us what subsequently became of them; that "the nerves were squeezed together," and "the spinal marrow pressed upon," and "the vertebræ thrust out of their place," without being accompanied by a statement that such accidents were followed by convulsions, paralysis, or death, as ancient and modern authors teach us to believe; but are mentioned as common occurrences in modern times, of no great importance or serious consequence.

It is not intended to deny that relaxation of the ligaments of some joints occasionally takes place: on the contrary, it has been already advanced in this essay, that such is not of unfrequent occurrence in the ankle-joints of children, by which their extent of rotation is greatly increased, and the patients are often rendered incapable of walking on the flat sole of the foot, but are under the necessity of walking on one side of it, (more particularly on the inside;) and Sir A. Cooper has lately recorded three instances of dislocation of the patella from the same cause: but, unless future facts and experience shall convince us of the contrary, we shall distinctly deny that either permanent curvature of the spine or luxation of the vertebræ of the spinal column is caused by relaxation of the vertebral ligaments. First, because the joints of the spine are secured, not by one, but by many ligaments; and dissection has not yet disclosed to us that relaxed condition of the vertebral ligaments, which can be justly considered the cause of curvatures of the spine, or of the vertebral "bones not holding together." In our own dissections it has not been observed; and in the dissections of Dr. E. Harrison's two cases which have been recorded, (and they are the only two,) no such appearance is mentioned, as will be plainly evinced by referring to the morbid examination of Colonel Sibthorpe by Mr. J. Swan,* and of Miss R. S. by Dr. E. Harrison, in No. 291 of this Journal. Indeed, in the long detail of Miss R. S.'s case, the word ligament never occurs, and seems to be alluded to in the following passage only: "Neither the vertebræ themselves, nor *their involucra*, participated in the least: they continued *sound*, or only suffered externally from the corrosive quality of the pus resting upon

---

* *Observations on the Nervous System*, by J. Swan.

them." Dissection sometimes discloses a state of the ligaments, which is the consequence, and not the cause, of the curvature. Thus, on the convex side or outer line of the curve, the ligaments are stretched and elongated, and consequently made tense; whilst, on the inner line or concave side of the curve, they are shortened or contracted in a ratio corresponding with the diminished thickness of the vertebræ involved in the curvature; and, should the bodies of some vertebræ be wholly absorbed, without the anterior ligaments being destroyed, the ligament that extends from the vertebræ above to those below is shortened in the inner line of curve, and only lies loose when the spine is most bent, and then the ligaments on the outer line of curve are stretched and tense. But it is contended that a relaxed condition of the vertebral ligaments, which will admit of the vertebræ being moved from side to side or backwards and forwards, has most assuredly not been witnessed as "a common cause of the common variety of curved spine." Much stress has been laid on the opinion of Dr. Glisson, contained in the following quotation: "Hinc sæpe fit, ut ligamenta vertebrarum spinæ a parte lateris frequentius prominentis laxentur atque elongentur, a parte vero opposita contrahantur; ita ut tractu temporis secundum rectam et naturalem lineam spina erigi non possit."* No proposition, however, can be more mathematically plain, than if a ligament of "*inelastic*" matter be elongated, it must be stretched, and therefore placed in a tense, and not a relaxed state; and this being the precise condition of the ligament on the convex side of the curve, it renders the expression of relaxed and elongated incompatible.

Secondly. Let it be admitted, for argument's sake, that relaxation of ligaments does exist, and allows of an extensive play and motion of the vertebræ on each other, still it would not necessarily follow that such a state should be the cause of spinal curvature: for it has been clearly demonstrated that no permanent curvature of any part of the spine can be induced, as long as the bodies of the vertebræ and intervertebral substances preserve their natural dimensions; and this they could and must do, if the disease be confined to relaxation of ligament.

Thirdly. If relaxation of ligaments were the cause, it would naturally result from the superadded powers of increased and extended motions of the vertebræ, the same as is observed in the ankle-joint, that, if the bones could be displaced and curved with facility, from the ligaments not holding them together, they could also be at once replaced in their natural line with ease by mechanical force, and the crooked soon made straight. But the fact is, we have never seen a case of *permanent curva-*

* *De Ruchitide*, p. 131, Ed. secund.

*ture* of the spine, in which the projecting and arched bones could be reduced at once to their proper spinal line by any force or means whatever, nor a case that gave satisfactory evidence of relaxed ligaments and loosened vertebræ.

Fourthly. When several of the bodies of the vertebræ are destroyed by progressive or ulcerative absorption, what powers prevent the spine from separating or dividing at this point? or, to adopt the phraseology of the abettors of the doctrine we impugn, what holds the remains of the half-destroyed bones together? The answer must be, there is no other competent power than the ligaments, which are somewhat assisted by the muscles: but it is a well-known fact, that the muscles will not hold a joint together after the ligaments are rendered incompetent, or are destroyed. The ligaments are first assisted by the muscles, and subsequently much more so by the anchylosis of the spinous processes, described in Jane Archer's case.

Having denied the premises, it follows that we cannot admit the consequences, and reject the easy transitions from relaxed ligament as a cause, to luxation and subluxation of the vertebræ as effects: 1st, because neither dissection discloses, not even in Dr. E. H.'s own cases, nor do the preparations in the anatomical museums, display either of those states after death. 2dly. Because we have not seen either state of luxation in any case of distorted spine in the living. 3dly. Because we are sceptical enough to believe that complete luxation of the spine must necessarily be followed by death, notwithstanding Dr. E. H. states that, on examining his patient, F. G. Pratt, of Camden Town, ten months after the accident occurred, he "found the first lumbal vertebra *wholly dislocated*, and driven into the left loin. The last dorsal and second lumbar were also displaced,"* &c.; and subluxation must be followed by pressure on the spinal marrow, producing paralysis of the parts inferior, convulsions, and even death. Indeed, if relaxed ligaments admitted of the extended play of the vertebræ on each other, we might induce paraplegia, and remove it at our pleasure, by causing subluxation and pressure on the spinal marrow. 4thly. The best and greatest authorities, Sir A. Cooper, Pott, and Boyer, declare there is no dislocation. 5thly. In those cases where several of the bodies of the vertebræ are destroyed by progressive or ulcerative absorption, and in which the dislocation of the processes would be the easiest, luxation has not been induced, or at least not recorded; but nature, always wise, always preservative in her efforts, prevents such a result by forming an

* See *Med. and Phys. Journal* for December 1820, p. 449. The subsequent history of this patient's case would be valuable, as we have only one report of it, which has not been continued; and, in those days of improvement, a dislocated back of ten months' duration may have recovered!

anchylosis of the spinous processes, that must be broken before any dislocation can ensue.*

The doctrine of relaxed ligaments being expressed in the following summary manner :—That the action of the dorsal muscles elongates and relaxes the vertebral ligaments; the relaxation of ligaments occasions luxation; and dislocation causes permanent curvature and distortion. It must be observed, that reason and experience reject them, because they are not drawn from the great book of nature, but appear to be deduced from assumed notions, that can scarcely be allowed to be possibilities.

There are, however, some changes produced in ligaments in this disease, besides those which have been cursorily described.† In cases of excurvation of the spine, proceeding from caries or ulcerative absorption, the ligamentum anticum commune and ligamenta intervertebralia, covering or attached to the bodies of the vertebræ which are carious, suffer in the general havoc, and are involved in the ulceration as far as the boundaries of the caries extend: hence, on dissection, those ligaments are discovered to have been, by the different stages of inflammation, thickened, increased in vascularity, ulcerated, and destroyed by ulceration; but, as the caries very rarely extends to the processes of the vertebræ, and not frequently to the posterior portion of the bodies, the ligamentum posticum commune, the ligamenta interspinosa, intertransversalia, and capsularia, are preserved free from disease, and continue to hold those portions of the vertebræ together, and secure the spinal column from dislocation and fracture; an important office, in which they are in some degree aided by the muscles, and subsequently by anchylosis of the spinous processes, as already stated.

In the proportion that experience and reason have induced us to reject the theory of a relaxation of ligaments being an ordinary cause of temporary, or, if a cause at all, of permanent curvature of the spine, do they confer authority for extending the limits of muscular debility as the cause of both. When the reasoning faculty is engaged in maintaining a particular theory or favourite hypothesis, it is equally amusing and curious to observe with what an accommodating spirit it overlooks many facts, and with what facility it bends others to its purpose: hence it is that some will not allow either temporary or permanent curvature of the spine to be attributed to the lost power of the dorsal muscles, and have exhausted some ingenious speculations in support of their dissent. As, in the investigation here insti-

* In the case of a child in North-street, Marylebone, whom we occasionally visited, (although the parents refused surgical assistance,) and who died very suddenly, we had some suspicions of this accident having occurred, but had no opportunity of ascertaining it.

† See p. 93.

tuted, we have prescribed to ourselves a rule to be guided by facts rather than by theories, or the authority of deservedly imposing names, and by legitimate deductions rather than preconceived notions, so facts compel us to infer that muscular debility is an immediate cause of temporary curvatures; and, by leading to habitually bad positions of the body, is the predisposing cause of permanent curvature of the spine. It is also clearly demonstrable, that a general muscular debility induced by a general cause, such as fever, measles, &c., which necessarily involves the dorsal muscles, frequently occasions relapses by increasing the curvature, where it has previously been in a state of progressive improvement. The dorsal muscles are, perhaps, weak in all cases, but they are more particularly so in others.

The muscular debility may be innate,—that is, exist from the birth; or the power of the muscles may have been fully developed, and subsequently diminished or lost. The former is observable during the period of infancy, the latter during childhood and adult age. Weakness is a relative term, generally applicable to loss of power in the muscles of volition. This debility may be partial, and limited to a set of muscles adapted to one particular purpose or the movements of a particular part of the body; or it may be general, and involve the whole of the voluntary muscles, as during or after a debilitating disease. The effects of partial weakness from the birth are more strikingly, if not more commonly, evinced in the lower extremities; as little patients are frequently presented, who have not had any use or power of the muscles of the extremity, or one leg, from the birth; the result of which is, that such extremity never grows in the proportion of its fellow, and is diminutive in size, paralytic, and useless. Such may be seen hopping about the streets on crutches. The patients who exhibit debility of the dorsal muscles in childhood, frequently display marks of incomplete formation of some of their bones: thus we have seen, in such cases, the shafts of the bones of the extremities very small; and, in two instances, the clavicles have been divided into two portions, united by something like ligament, that by degrees became ossified.

Where the muscular debility has not been innate, it has been induced by diseased brain, rheumatism, or the general weakness ensuing from dentition, fevers, or any of the exanthemata.

When the dorsal muscles are, from weakness, inadequate to sustain the spine in an upright position: if the patient attempt to assume the erect attitude, or to sit upright, the whole of the spine is bent, in some cases, from the "straight" spinal line into the form of a regular bow, or "the shape of a half-bent hoop," just such as the part would assume when any one voluntarily curves the back to a great extent, by bending forwards and projecting the lower dorsal vertebræ backwards; a posture in which all the vertebræ would be more or less implicated in the curvature. Three cases of this slight variety of temporary curvature have been recorded as they occurred in the adult, and only one of its being met with in childhood;* yet it is by no means of rare or infrequent occurrence among children and minors. In whomsoever this variety appears, the curvature in the early stages is, at all events, only temporary, and the spine can be restored to its natural line by placing the patient on his back on a horizontal plane, and desiring him to draw up and bend the thighs on the body; or it can be effected with more accuracy and certainty by laying the patient in the facial horizontal position, and employing, in aid of position, gentle mechanical pressure on such points or processes of the vertebræ as remain at all prominent. It has been affirmed by Mr. Baynton, that no one of the vertebræ in this variety projects more than another, but that the spine appears just as if it were bent forwards moderately by the will. If any one, however, will take the pains to examine the back with attention and accuracy, he will perceive, in general, either one or three spinous processes project more than the others from the middle of the curve, which has necessarily become the point of pressure, on the inside of the curve, and of greatest separation and extension on the outside; and that the skin over these processes is stretched, and appears whiter than over the other processes, which generally display no mark of particular projection.

It has been also assumed, that this variety of *temporary* curvature, arising from muscular debility, necessarily involves *all* the vertebræ: but this by no means accords with our experience, as several cases of children have been presented for advice, where the curvature has been limited to three or four of the lower dorsal and one or two of the upper lumbar vertebræ, or to the dorsal vertebræ between the scapulæ, in which the spine could be reduced to its natural line, in the manner above mentioned. In these cases, the excurvation seems to be produced by the infant's being placed too much in the upright posture of the back, before the muscles have sufficient power, or the vertebræ adequate firmness, to sustain it. Let it then be admitted, if a patient, with an excurvation of the spine in the erect attitude, regain the natural form of the back on being placed in either the dorsal or facial horizontal position, with or without the aid of a gentle mechanical pressure, that the curvature, from being removable at will, can only be denominated

---

* One by Mr. BAYNTON, in his Essay; and the others by the ingenious Mr. H. EARLE, in the 41st Number of the *Edinburgh Med. and Surg. Journal.*

temporary; and that its cause essentially consists in the inherent want, or subsequent loss, of power in the dorsal muscles. At an early period of this form of disease, no structural change takes place in the vertebræ, and none would ensue, if proper means of prevention were employed; but, should the patient be neglected, the structural changes, which lead to permanent curvature, will sooner or later be induced, however it may be deferred for months, or even years, when any circumstance has caused the patient to be confined more than usual to the recumbent position.

An endeavour will be made, by and by, to explain in what manner this deficiency of muscular power becomes a predisposing cause of permanent curvature; having witnessed the gradual operation of this cause, and the progress of its effects from temporary curvature or projection of some vertebræ to a permanent and complete excurvation; and the parents of some young persons thus permanently affected have, with much apparent accuracy, traced the origin to so early a date of existence as three months, and have been able to describe the increasing progress and gradual effects of the disease from that period up to manhood.

A description of the course of the disease will now be attempted in such cases as have arisen from muscular weakness from the birth, and which has prevailed in the subsequent growth of the body, from no successful means being employed to arrest it. In this case, as soon as the infant's age admits of a trial being made to set it upright, the back may be observed to bow outwards; and, although the whole of the spine be more or less curved, yet, after a short time, it is generally apparent, on a strict examination, that three or four of the lower dorsal, with one or two of the upper lumbar vertebræ, project a little beyond the regular line of curve. As the spine recovers its "straight" position at this period, when laid in the horizontal posture, an alarm or suspicion of an incipient distortion is not always created. While the infant advances in age and size, the mother is surprised to find that this muscular debility, or "weakness of the back," (as she expresses it,) continues, and that he does not acquire the use of the lower extremities so early as infants usually begin to walk. The period of dentition having now arrived, the continued debility is ascribed to the exhausting effects of the irritation from cutting the teeth, and apprehension is lulled to sleep, until dentition be completed, and the inability of using the lower limbs still remains. After, and perhaps during, dentition, trials are instituted to make the child walk; but these frequently fail, and occasion the child to cry. In the worst cases, paralysis of the lower extremities assumes a permanent form: children, however, do not generally lose the entire use of the lower extremities, but, after an uncertain lapse of time, they acquire the power of walking, (although with less strength and activity than healthy children,) by the aid of such supports as stand in the room. In infancy, the infant is necessarily much confined to the horizontal posture, during which the curvature, observable in the sitting position, is reduced to and retained in its natural line; a circumstance that arrests the progress of disease, or only allows it to increase with a very slow gradation. As the child advances in age, it sleeps less and sits up more, and the excurvation increases faster. When the child can walk about, the progress of the curvature is more rapid; and the spinous processes, in the erect attitude of the patient, project, and exhibit the shining and stretched appearance of the skin over them. Walking firmly and uprightly for more than a short period, cannot be accomplished; but the little patient continues to avail himself of the artificial aid of the chairs and tables in the room, or of a pair of small crutches. Should he endeavour to walk without any support, his legs will perhaps cross, his steps may be unstable and imperfect, or his feet may strike against something uneven; and in either case he will fall. In the progressive growth of the body to maturity, a more defined excurvation of the spine is induced; and it is most common for six, eight, or the whole of the dorsal vertebræ to be more particularly involved. As the bodies of the dorsal vertebræ become absorbed, and the curve of the back increases, a gradual change is induced in the natural form and shape of the ribs inserted into them. The contour of the bones of the chest nearly approaches that of a circle; there is, however, a little more breadth than depth; but, as the vertebræ are protruded backwards, they necessarily lengthen and draw out the vertebral ends of the ribs, which now project beyond and between the scapulæ, and cause them to assume a lateral situation; the sternum is thrown forwards, and carries out the sternal ends of the ribs, so that the chest is reduced to an oval shape, and the ribs are said to be flattened on their sides: thus the natural dimensions of the chest are reversed, and there is more depth than breadth, and the patient becomes "chicken-chested" and "hump-backed."* During the increase of the curvature, either the horizontal surfaces or nearly the whole of the bodies of the vertebræ are removed by absorption; some of the spinous processes are separated at a greater distance than natural, and the calibre of the spinal canal is diminished at the centre of the curve. In this state of things, the dorsal muscles around the most projecting points appear smaller and paler than usual, and are probably weaker, and the

* See Miss J. Archer's case.

patient supports himself in walking and sitting by placing the hands on the knees. It sometimes happens that the curvature becomes double, the lumbar vertebræ being the seat of the second curve; and, before the growth of the body has been finished, we have seen instances of the spine exhibiting a double deviation from the natural line, by a lateral curvature being superadded to the excurvation. In the mean time, the subject of this lamentable deformity is stunted in all the stages of its growth, feeble in structure, thin, pale, and emaciated. If a disposition to scrofula exist, marks of it are sooner or later made evident. The patient is generally afflicted with pains across the epigastric region, and disordered digestive organs and kidneys; and, after the chest becomes deformed, is rendered subject to dyspnœa, asthma, pneumonia, and cough. The following appears to be the succession of effects on the spine:—Debility of the dorsal muscles occasions the upper parts of the body to incline forwards, and the spine to curve backwards, as the muscles cannot sustain it in the erect attitude; the forward inclination of the body and superincumbent weight cause an unnatural pressure on the anterior portions of the bodies of the vertebræ; this pressure leads to their progressive absorption, and that of the intervertebral substance; which produces permanent excurvation of the spine, by giving a cuneiform shape to the bodies of the vertebræ, and making them thinner on the anterior than the posterior part, (if they do not become wholly absorbed,) so that the bones, placed on each other, cannot be made to assume a perpendicular elevation or erect attitude, but must arrange themselves into the form of an arc or curve.

CASE V.

H. Peckman, æt. five, 1, Brown-street, Bryanston-square, on the 7th of March was admitted a patient of the Marylebone station of the Royal Metropolitan Infirmary for Diseases of Children. He had for some months been gradually declining in strength, more particularly in the back. He soon become fatigued; and, from inability to walk upright, he was generally induced to sustain his body, in his movements about the room, by placing his hands on the chairs, tables, &c. that stood in his way. He complained of pain and tightness across the epigastric region. His bowels were regular. On examining the spine, a slight projection of the spinous processes of the fourth, fifth, sixth, and seventh dorsal vertebræ, and of the two upper lumbar, was observable, which, however, scarcely deviated from the spinal line; and, on laying the patient in the facial horizontal position on a feather-bed, and pressing with a gentle mechanical force on the projecting processes, they were, to all appearance, replaced in their natural situation, and the pressure occasioned very little pain. In this incipient state of distortion, no perfect arc is yet formed, but the existing derangement may be recognised by the spinous processes stretching the skin over them, and making it appear white and shining, resembling the phenomena of the stretched skin over the knuckles of a clenched fist. A compress, pad, shield, and bandage, were applied, and the facial horizontal position enjoined.

The patient continued under this plan of treatment three weeks, and had evidently improved in strength and in the appearances of the back, when he was infected by measles, the febrile stage of which was extremely severe, and induced very great general debility, of which the dorsal muscles partook. As the mechanical management of the spine had been laid aside during the measles, and as the patient had been subsequently allowed to sit up, and follow the bent of his inclinations in his movements, with a view of restoring his general health, the spinous processes were soon projected more than when first seen, and he was again confined to the treatment commenced and employed before the attack.

Of the confinement to bed indispensable to our plan of treatment, and founded on the principles by which it is conducted, most children are impatient, and of its propriety many parents cannot be made sensible; both of which circumstances occurred in the present case, and after the boy had submitted again to the practice about a fortnight, we found him sitting on the staircase, and inquired how he came to be there? "Oh! (said his mother to Mr. Seynage and me,) his father did not like his confinement, so we took further advice. We carried him to Dr. Latham, (sen.) who told us to take off all his apparatus, and that confining him to bed would make him more ricketty than he was: therefore, said the Doctor, let him run about, and send him to the country for six months, where he will get strong and hearty, and the back will not become crooked." As Dr. L. had written on the subject of curved spine in the year 1795,* and had "this goodly seeming" of having directed his attention to the subject, the opinion of this experienced physician occasioned us to doubt the correctness of our own, and to imagine that this case possibly might not be an incipient one of curved spine; although our experience and observation had induced us to conclude that it was so, and to treat it as such.

The boy was sent to Hammersmith in June, and the repeated accounts received from the lady who recommended him to our care informed us that the excurvation of the spine was gradually

---

* See a Letter of Dr. L.'s, in Sir JAMES EARLE's Observations on the Curved Spine.

increasing, and, in spite of country-air, both local and constitutional health had suffered much. He returned home at Christmas; and, in April, I was again requested to visit him, at 26, Ceymour-place. He was much thinner, and very weak; his appetite was bad; the pain across the epigastric continued; and the double curvature of the vertebræ was completely established, of which variety he indeed exhibited a good specimen. The chest now assumed an oval form; eight of the dorsal vertebræ formed a curve that projected backwards between the scapulæ, which were thus thrown in a lateral situation; the sternum projected forwards; the ends of the ribs were drawn out both behind and before, and the chest assumes an oval form; the cartilages of the false ribs advanced into the epigastrium, and approximated so near that the opposite sides almost touched. The sternum inclines to the right side, and appears twisted. He could only walk in the bent posture, and not any length of time without experiencing fatigue, and stopping to take rest. His bowels were regular. He had always an eruption of sores on the skin. An offer was now made to undertake the cure, provided the objectionable horizontal position would be considered a *sine qua non*, and be submitted to. On the next visit, we were informed that an oilman, of the name of Ritchie, at Westminster, promised to effect a cure by plasters and the steam-bath, without enjoining a recumbent posture, and our services were withdrawn. Ritchie's quackery was discovered in three weeks; and, in May, the child was carried to Mr. J. Brookes, who urged the propriety of a recumbent posture, and ordered some purgative, that has brought away some black fæces, and improved the general health. On the 17th of June, being again requested to visit the child, it was found that a lateral curvature was added to the other deformities, and the patient was very much emaciated. The boy now began to submit to the facial horizontal posture, and is certainly improving.

This case evinces the uninterrupted progress of the disease, and proves the necessity of professional assistance in the earliest stage. It also shows how readily a relapse, or increase of curvature, follows any acute disease that induces general debility.

The essay will be discontinued at present, in the hope that the author will be enabled to lay it before the public in a more connected form in a few months. In the mean while, he would feel particularly indebted to any one, who will afford him opportunities of being present at dissections of diseased spines, or of visiting interesting cases.

37, *Bedford-street, Covent Garden;*
*June 30th,* 1823.

